# Surviving Emphysematous Gastritis after Hepatectomy

**DOI:** 10.1155/2013/106383

**Published:** 2013-03-14

**Authors:** Harry Hok Yee Yu, Simon Tsang, Tan To Cheung, Chung Mau Lo

**Affiliations:** ^1^Department of Surgery, The University of Hong Kong, 102 Pokfulam Road, Hong Kong; ^2^Department of Surgery, Queen Mary Hospital, The University of Hong Kong, 102 Pokfulam Road, Hong Kong

## Abstract

Emphysematous gastritis is a rare variant of phlegmonous gastritis due to invasion of stomach wall by gas-forming bacteria. It is characterised by abnormal presence of gas in the stomach by imaging, in association with clinical sepsis. Patients suffering from this condition usually present with an underlying pathology. We are reporting a middle-aged Chinese male with hepatitis B virus related hepatocellular carcinoma. He underwent partial hepatectomy and was diagnosed with emphysematous gastritis 4 days after index operation. Emergency laparotomy, including upper endoscopy, was performed. He was managed with antibiotics and discharged 18 days after second operation. This paper shows a review of the literature about the disease, with particular attention to pathology, clinical features, and management.

## 1. Case Report

**Figure 1:**
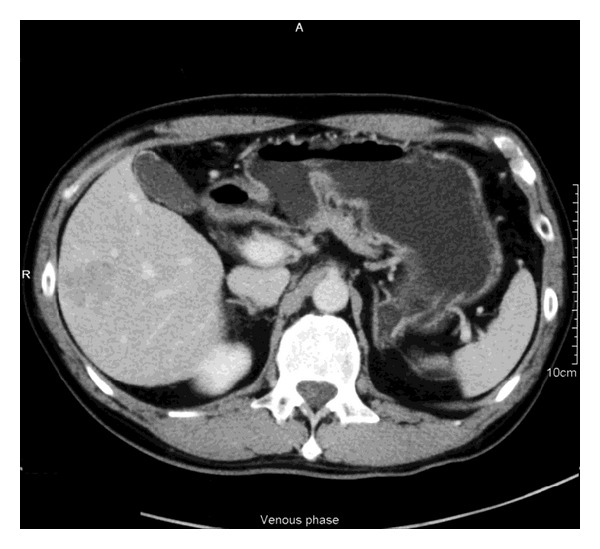
Preoperative CT scan showing the hepatocellular carcinoma at segment 6. The stomach was unremarkable. No gas was noted in the portal vein.

A 52-year-old man was known to have hepatitis B virus related cirrhosis. He was a nondrinker and otherwise enjoyed good past health. He had regular ultrasound screening of his liver. A 3 cm lesion was detected by screening ultrasound in the right lobe of liver. Subsequent contrast CT abdomen confirmed the presence of a 3 cm lesion in segment 6 with arterial enhancement and early portovenous washout (Figure 1) suggestive of hepatocellular carcinoma. His complete blood picture was normal. Serum bilirubin level was 24 *μ*mol/L. The serum albumin level was 36 g/L. Open wedge resection of the liver tumour was performed. The operation lasted 4 hours, and blood loss was 200 mL. No blood transfusion was required perioperatively. Pringle maneuver was not performed throughout the procedure. A nasogastric tube was placed in the stomach for decompression. Amoxicillin/clavulanic acid 1.2 g and esomeprazole 40 mg were administered intravenously immediately before operation and were continued postoperatively. Histopathological examination confirmed well-differentiated hepatocellular carcinoma with closest resection margin 1 cm.

**Figure 2:**
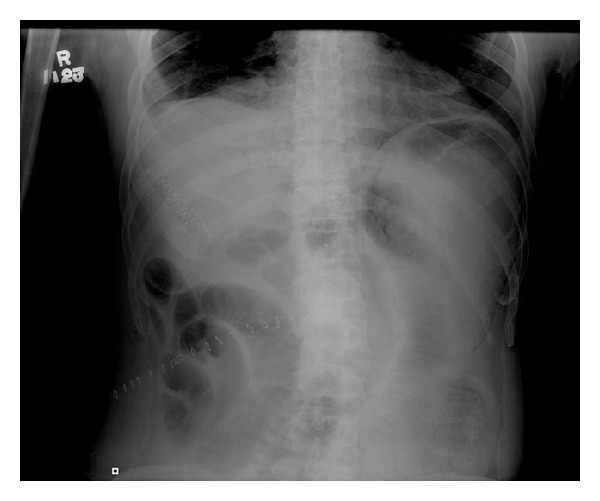
Abdominal radiograph on postoperative day 4: lucency in the stomach and small bowel ileus.

**Figure 3:**
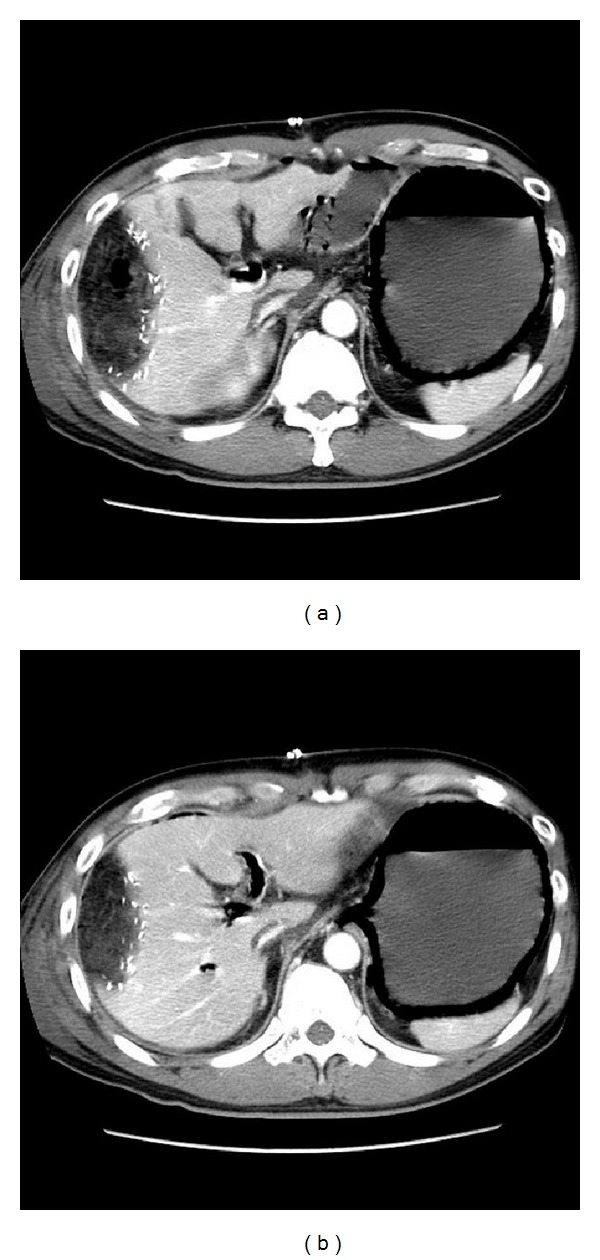
CT image confirming presence of gas in stomach wall. Gas is also noted in the portal vein.

Nasogastric tube was removed on day 1 and diet resumed on the next day. However, patient complaint of shortness of breath, palpitations, and epigastric discomfort on post-operative day 4. Physical examination revealed tachycardia (130 bpm) and mild epigastric tenderness. Blood tests showed neutrophil predominant leukocytosis and bilirubin raised up to 45 *μ*mol/L. Arterial blood gas showed metabolic acidosis (pH 7.24, HCO_3_^−^ 16 mmol/L and base excess −6 mmol/L). Erect abdominal radiograph showed dilated small bowel and a lucency of gas within the gastric wall (Figure 2). Urgent contrast enhanced CT abdomen was performed, which showed extensive gas in the submucosal layer of his stomach and gas in his portal vein (Figure 3).

**Figure 4:**
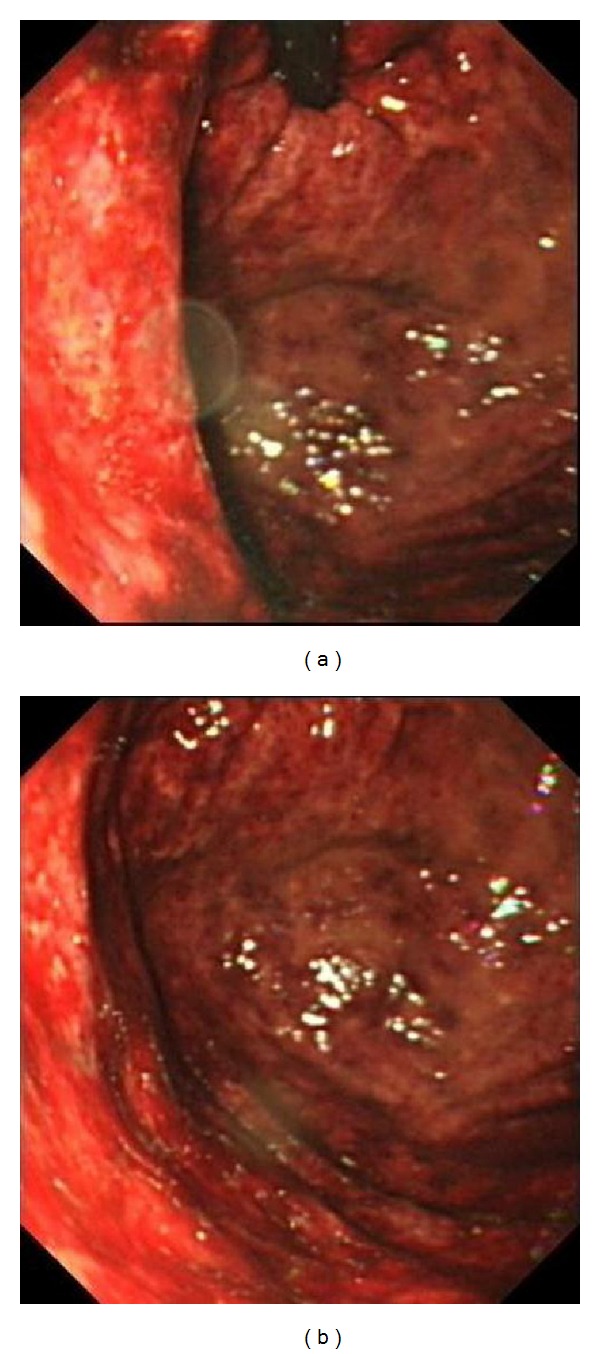
Intraoperative upper endoscopy showed congestion of gastric mucosa.

The patient underwent exploratory laparotomy immediately. The peritoneal cavity was clean. The liver was not congested and no bile leakage was identified. Intraoperative ultrasound demonstrated gas in his portal vein, but the blood flow was good. The serosal surface of stomach was healthy, but crepitus was noted in the entire stomach. On-table upper endoscopy revealed congestion of gastric mucosa with no evidence of gangrenous change (Figure 4). His duodenum and proximal jejunum were unremarkable. Gastrectomy was not performed in view of the absence of solid evidence of gastric gangrene or perforation. He was transferred to the intensive care unit for close monitoring after the first operation. His antibiotic was stepped up to piperacillin/tazobactam after the second operation in view of recent antibiotic exposure. Total parenteral nutrition was initiated. Tissue culture of his gastric mucosa yielded *Klebsiella pneumoniae*. Histopathology of the gastric mucosa confirmed ischaemic changes. 

Healthy gastric mucosa was seen on upper endoscopy performed 7 days after second operation. Diet was resumed on the same day, and the patient was discharged 18 days after the second operation.

## 2. Discussion

The finding of gas within the wall of the stomach is always alarming. Such an observation was made in 1889 by Fraenkel [1]. While gastric emphysema is a benign condition due to gas entering the stomach wall through a mucosal defect, emphysematous gastritis is due to an infective organism producing gas within the stomach wall. This pathological condition, regarded as a severe bacterial infection of the gastric wall, carries a mortality rate ranging from 41% to 60% [2–4]. 

Patients usually present withan underlying pathology with clinical sepsis and abdominal symptoms such as diarrhoea, nausea, and vomiting [3]. Abdominalradiograph is the simplest clinical investigation, which may demonstrate irregular strips of gas within the wall of the stomach. Computed tomography, however, is the best modality to establish the diagnosis. Radiological features of this condition in the CT scan include layering of gas bubbles, wide streaks, or a cystic pattern of lucency within a thickened gastric wall [5]. Endoscopic examination of the stomach may reveal absence of mucosal folds, mucosal edema, or necrotic mucosa in the stomach [3]. In theory, the stomach is rarely affected by severe inflammation due to its unique anatomy including a good mucosal barrier, acidic pH, and an excellent blood supply. However, any antecedent injury may predispose a patient to erosion of the gastric wall and its septic complication [5]. These patients are usually immunocompromised or immunosuppressed. The more common causes included underlying malignancy, use of systemic immunosuppressant, extensive burn, diabetes mellitus, surgical stress, liver failure, and renal failure [3, 5–8].

The salient features of gas within the gastric wall in emphysematous gastritis could be due to bacterial overgrowth as may be shown by increased hydrogen levels in the gastric mucosa samples [4]. Theoretically, any gas-forming bacteria can cause this condition. Commonly seen pathogens include *Escherichia coli*, *Enterobacter* species, *Pseudomonas aeruginosa*, *Clostridium species*, and *Staphylococcus aureus* [4, 5]. *Clostridium* infection could lead to clostridial shock resulting from release of inflammatory mediators by these toxins. The release of cytolysins results in leukostasis, thrombosis, and tissue hypoxia.

Gastrectomy is indicated in patients refractory to medical therapy or when complications arise. Indications for emergency gastrectomy include clinical deterioration despite optimal medical management, perforation of the stomach and gastric infarction [5]. Additional second stage reconstruction may be required in 21% of patients who suffered from esophageal stricture after ingestion of corrosives [4].

Gas gangrene of the stomach can lead to fatality after liver resection even in a healthy individual, as reported by a tragic death of a living liver donor in 2004 [6]. Temporarily occlusion of the portal venous inflow may predispose the alimentary tract to ischaemia. Anaerobes in the gut flora may overgrow during this period. The release of the portal blood flow after hepatectomy may lead to an overwhelming circulation of these bacteria. The use of potent proton pump inhibitors for ulcer prophylaxis in major hepatectomy may also lead to a suppression of the resident gastric flora leading to an overgrowth of gas-forming organisms. Although the natural clinical course of this condition is not well studied and a high mortality rate is anticipated [3], prompt diagnosis and treatment should improve survival. With a degree of clinical suspicion, emphysematous gastritis should be detected early. A simple abdominal radiograph may suggest the presence of this condition. Once the condition is suspected, the patients should be treated as severe clinical sepsis. Aggressive resuscitation with intravenous fluids with broad-spectrum antibiotics covering Gram-positive, Gram-negative, and anaerobic bacteria should be administered promptly [3–5]. In our scenario, *Klebsiella pneumonia* was the only identifiable organism which was known to be a common gas-forming entity in patients with liver abscess [9]. *Piperacillin/tazobactam *was employed in this case because it has a good coverage against Gram-negative bacteria as well as anaerobic bacteria such as *Clostridium perfringens. *Total parenteral nutrition is usually required as absorption is initially inhibited, due to mucosal ischaemia [10]. Reversible underlying condition such as possible surgical complications should be corrected with vigorous treatment including surgery. Good blood sugar level monitoring by cautious use of insulin may improve survival [9].

To conclude, gas-forming gastritis following liver resection is usually fatal. Vigilant postoperation care, monitoring, and high index of suspicion for this condition can ensure prompt treatment and optimize survival.
